# Text Messaging and Mobile Phone Apps as Interventions to Improve Adherence in Adolescents With Chronic Health Conditions: A Systematic Review

**DOI:** 10.2196/mhealth.7798

**Published:** 2017-05-15

**Authors:** Sherif M Badawy, Leonardo Barrera, Mohamad G Sinno, Saara Kaviany, Linda C O’Dwyer, Lisa M Kuhns

**Affiliations:** ^1^ Ann & Robert H. Lurie Children's Hospital of Chicago Department of Pediatrics, Division of Hematology, Oncology and Stem Cell Transplant Northwestern University Feinberg School of Medicine Chicago, IL United States; ^2^ Zagazig University Faculty of Medicine Department of Pediatrics, Division of Hematology and Oncology Zagazig Egypt; ^3^ Ann & Robert H. Lurie Children's Hospital of Chicago Department of Pediatrics, Division of Hematology, Oncology and Stem Cell Transplant Chicago, IL United States; ^4^ University of Kansas School of Medicine Department of Pediatrics Wichita, KS United States; ^5^ Advocate Children's Hospital Department of Pediatrics Oak Lawn, IL United States; ^6^ Northwestern University Feinberg School of Medicine Galter Health Sciences Library Chicago, IL United States; ^7^ Ann & Robert H. Lurie Children's Hospital of Chicago Department of Pediatrics, Division of Adolescent Medicine Northwestern University Feinberg School of Medicine Chicago, IL United States

**Keywords:** adolescents, medication adherence, chronic health conditions, chronic medical conditions, text messaging, mobile phone apps, smartphone apps, smartphone applications

## Abstract

**Background:**

The number of adolescents with chronic health conditions (CHCs) continues to increase. Medication nonadherence is a global challenge among adolescents across chronic conditions and is associated with poor health outcomes. While there has been growing interest in the use of mHealth technology to improve medication adherence among adolescents with CHCs, particularly text messaging and mobile phone apps, there has been no prior systematic review of their efficacy.

**Objective:**

The purpose of this review was to systematically evaluate the most recent evidence for the efficacy of text messaging and mobile phone apps as interventions to promote medication adherence among adolescents with CHCs.

**Methods:**

PubMed, Embase, CENTRAL, PsycINFO, Web of Science, Google Scholar, and additional databases were searched from 1995 until November 2015. An additional hand search of related themes in the Journal of Medical Internet Research was also conducted. The Preferred Reporting Results of Systematic Reviews and Meta-Analyses guidelines were followed. Two reviewers independently screened titles/abstracts, assessed full-text articles, extracted data from included articles, and assessed their quality using Grades of Recommendation, Assessment, Development, and Evaluation criteria. Included studies were described in original research articles that targeted adherence in adolescents with CHCs (12-24 years-old).

**Results:**

Of the 1423 records examined, 15 met predefined criteria: text messaging (n=12) and mobile phone apps (n=3). Most studies were performed in the United States (11/15, 73%), were randomized-controlled trials (8/15, 53%), had a sample size <50 (11/15, 73%), and included adherence self-report and/or biomarkers (9/15, 60%). Only four studies were designed based on a theoretical framework. Approaches for text messaging and mobile phone app interventions varied across studies. Seven articles (7/15, 47%) reported significant improvement in adherence with moderate to large standardized mean differences. Most of the included studies were of low or moderate quality. Studies varied in sample size, methods of adherence assessment, and definition of adherence, which prohibited performing a meta-analysis.

**Conclusions:**

The use of text messaging and mobile phone app interventions to improve medication adherence among adolescents with CHCs has shown promising feasibility and acceptability, and there is modest evidence to support the efficacy of these interventions. Further evaluation of short- and long-term efficacy and cost-effectiveness of these interventions is warranted given the early and evolving state of the science.

## Introduction

In the United States, about 15-20% of children and adolescents have chronic health conditions (CHCs) (eg, asthma, diabetes), a number that has doubled in the past 20 years accompanied by increased health care expenses [1,2]. The increased rate of children and adolescents with CHCs is mainly driven by the rising prevalence of asthma and obesity, and the advances in medical care that have led to improved survival over time [3,4]. Adolescents with CHCs have specific health needs and contend with daily challenges involving their illness-related routine, including administration of daily or weekly medications, diet restrictions, lifestyle changes, laboratory monitoring, and outpatient follow-up with medical teams [5].

Medication adherence in particular is an important component of the treatment regimen as it often drives long-term outcomes. It is also a global public health problem, and it represents a barrier to achieving optimal health as a primary cause of treatment failure and avoidable complications [5]. Medication nonadherence rates are estimated to be 50-75% among pediatric patients with CHCs, with some evidence of lower adherence among adolescents [5,6]. Medication nonadherence has been associated with poor health-related quality of life (HRQOL) scores, increased morbidity and mortality, and increased health care utilization with an estimated US $100-300 billion of annual avoidable health care costs [6-13]. Engaging adolescents with CHCs in self-management skill building, including medication adherence, has long-term benefits [14-16]. Although treatment regimen and monitoring requirements vary across pediatric CHCs, most adolescents with CHCs perceived adherence barriers as multifaceted, but with common attributes across conditions [17].

Earlier systematic reviews and meta-analyses of pediatric patients with CHCs have shown evidence of a positive impact of interventions on medication adherence, HRQOL, and family functioning as well as reduction in health care utilization [18-24], although with primarily small effect sizes and methodological limitations. There has been growing interest in the use of technology to improve medication adherence and self-management skills in the last few years. Adolescents have ubiquitous access to mobile technology, in particular text messaging and mobile phone apps, across levels of social position and status [25-27]. The adoption of these technologies by adolescents has opened up new opportunities to link patients with their providers and to improve self-management and medication adherence. A recent review examined the efficacy of mobile apps in improving self-management skills, not specifically medication adherence, among adolescents with a physical CHC or long-term condition. However, they were not able to draw concrete conclusions because of the lack of evidence-based studies and the heterogeneity of the included studies [28].

The purpose of this review is to systematically evaluate the most recent evidence for the efficacy of text messaging and mobile phone app interventions in promoting medication adherence among adolescents with CHCs. We focused on text messaging and mobile phone app interventions in particular because these technologies are the most widely and frequently used by adolescents and are thus most likely to serve as the basis for future intervention development.

## Methods

We followed the guidelines for the Preferred Reporting Items for Systematic Reviews and Meta-Analyses (PRISMA) in the reporting of evidence across the studies we reviewed (Multimedia Appendix 1) [29]. This review was registered with the International Prospective Register of Systematic Reviews (PROSPERO) (CRD42015025907) [30].

### Article Retrieval

A librarian (LO) collaboratively developed the highly sensitive medical subject headings (MeSH) term‒based search strategies with other review authors (SB, LK) and from July to September 2015 ran searches in the following databases: PubMed MEDLINE; Embase; Cochrane Central Register of Controlled Trials (CENTRAL) on the Wiley platform; the Cumulative Index to Nursing and Allied Health Literature (CINAHL) (EBSCO); PsycINFO (EBSCO); Web of Science; Center for Review and Dissemination; and Inspec (EBSCO). Additional searches were run in November 2015 using the following sources: Proquest Dissertations; Scopus; ClinicalTrials.gov; World Health Organization Clinical Trials; Controlled-Trials.org; Institute of Electrical and Electronics Engineers Explore; and Google Scholar. Search strategies for all databases except MEDLINE were adapted from the PubMed MEDLINE strategy. All databases were searched back to 1995, which is a point in time when access to mobile phones began to increase rapidly. No language limits were applied. The search strategy specified keywords, including text messaging, phones, mobile apps, and portable software combined with adherence or compliance, and search terms related to child, pediatric, adolescents, and youth. We also reviewed the search strategies of previous studies to include additional terms. See Multimedia Appendix 2 for complete search strategies in each database. An additional hand search of related themes in the *Journal of Medical Internet Research* was also conducted. We also attempted to identify additional studies by searching the reference lists of key studies and relevant systematic reviews. We contacted authors of included publications to obtain additional studies meeting the inclusion criteria.

### Study Selection

The inclusion criteria were as follows: (1) adolescents (12-24 years old) [31] with a chronic illness requiring long-term daily or weekly medications for ≥12 months, (2) original research articles, (3) studies that were either randomized controlled trials, quasi-experimental studies, or pilot/feasibility studies (including single arm, pre-posttest), (4) text messaging or mobile phone‒based interventions (app or mobile intervention), and (5) medication adherence as the primary or secondary outcome. The exclusion criteria included (1) mean or median age of entire study cohort in the study was either <12 years old, >24 years old, or not specified, (2) adolescent participants were not the target of the intervention (eg, intervention targets babies born to adolescent mothers with human immunodeficiency virus or targets parents of adolescents), (3) text messaging and mobile phone apps as interventions focused on disease monitoring or ecological momentary assessment, but not meant to improve medication adherence, or (4) technology-based interventions other than text messaging and mobile phone apps, such as Web- or Internet-based interventions, personal digital assistant, etc.

### Data Extraction

We used a standardized form for data extraction. Data items in the extraction form included the following: first author’s name; publication year; country; condition or disease focus of the study; participants’ age; study design; duration of intervention and follow-up; components of text messaging or mobile phone app interventions; control group (if applicable); adherence measures; adherence rates; other outcome measures such as disease-related outcomes of morbidity and mortality, HRQOL, and self-efficacy or self-management skills; and theoretical framework. Two authors coded all included articles individually, and then the lead author (SB) independently reviewed all codes. Disagreements were resolved by discussion or by consultation with a third author, if needed.

### Quality Assessment and Strength of the Evidence

Studies described in each article were evaluated for the quality of evidence using the GRADE approach (Grades of Recommendation, Assessment, Development, and Evaluation) [32]. This method evaluates four different key domains including consistency, directness, risk for bias, and precision of the evidence. Two authors graded all included articles individually, and then the senior author (LK) independently reviewed all grades. Disagreements were resolved by discussion or by consultation with a third author, if needed.

### Data Analysis

Data were analyzed quantitatively and qualitatively. Our primary outcome measure was mean change in medication adherence rate, and data were analyzed for those who had baseline and follow-up values. We also analyzed mean change in adherence-related laboratory markers. Standardized mean differences (SMD) with 95% confidence intervals were calculated—using means and standard deviations—to evaluate the efficacy of text messaging and mobile phone‒based interventions in improving subjective and objective measures of adherence, including adherence rates and adherence-related laboratory markers [33]. Data were analyzed using Stata 13.

## Results

### Literature Search

**Figure 1:**
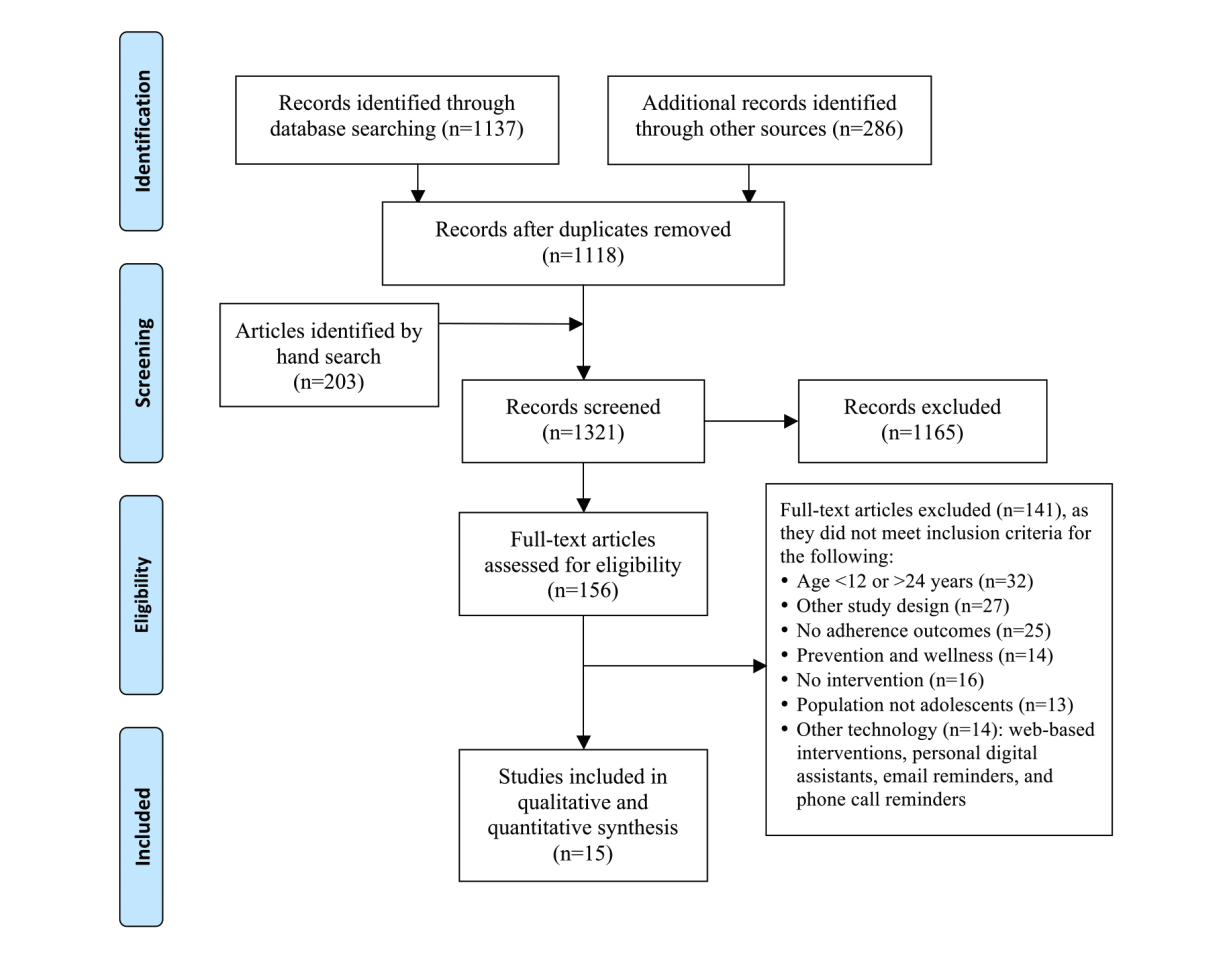
Flow of studies through the review according to PRISMA guidelines.

A total of 1423 citations were retrieved; 1137 in the July-August 2015 search and 286 in the search in November 2015. After removing duplicates, 1118 original articles remained (see Figure 1). Two authors (SB and LK) independently screened the article titles and abstracts of 1118 records against inclusion criteria, and a total of 156 records met all predefined inclusion criteria. Two authors (SB and LK) then independently reviewed the full text of these articles in detail against the exclusion criteria, and 141 articles were excluded. A total of 15 articles met all predefined criteria to be included in this review [34-48]. We did not identify any non-English articles that met our inclusion criteria. The study flowchart and reasons for exclusion of full text papers were documented in an adapted PRISMA study flowchart (see Figure 1) [49].

### Study Characteristics

Although our search included studies published since 1995, no eligible studies were identified before 2005, with most studies (12/15, 80%) published since 2010 (Table 1) [34-39,41-44,46,48]. Most studies were performed in the United States (in whole or in part; 11/15, 73%) [34,36-38,41,42,44-48]. Studies targeted eight different CHCs, including those of adolescents with acne (n=2) [34,39], asthma (n=1) [47], depression (n=1) [42], diabetes mellitus (n=4) [35,40,43,46], human immunodeficiency virus (n=2) [37,41], liver transplant (n=2) [44,45], sickle cell disease (n=2) [36,38], and systematic lupus erythematosus (n=1) [48]. Most studies were small in size (ie, ˂50 participants; 11/15, 73%) [34-37,42-48], and just over half (8/15, 53%) included samples of young adults (with a mean or median age ≥18 and ≤24 years old) [34,37,39,41-43,47,48]. In terms of study design, more than half were RCTs (8/15, 53%) [34,39-43,47,48], and the remainder were primarily single-arm nonrandomized trials (6/15, 40%) [35-37,44-46] or retrospective chart reviews (1/15, 7%) [38]. The duration of the studies varied: 2-4 weeks (2/15, 13%) [42,43], 12-16 weeks (5/15, 33%) [34,35,39,46,47], 24 weeks (3/15, 20%) [36,37,41], and 12 months or more (5/15, 33%) [38,40,44,45,48]. Only one study evaluated the durability of intervention effects in a crossover design with 6-month follow-up in one of the study arms after the intervention was discontinued [41]. Measures of medication adherence included self-report (9/15, 60%) or biomarkers (9/15, 60%), as well as other forms of monitoring (7/15, 47%), such as Medication Event Monitoring System caps, directly observed therapy, pill count, and pharmacy records abstraction. Three studies (20%) measured additional adherence behaviors, including completion of laboratory visits [44], clinic visits [48], and monitoring of peak expiratory flow values in patients with asthma [47]. Only four studies (27%) incorporated explicit theoretical approaches or frameworks into the model of intervention effects, including Theory of Planned Behavior [43], Gamification theory [35], and Social Cognitive Theory [40,41]. Most studies (12/15, 80%) were rated “low” or “moderate” according to the GRADE criteria (Table 1) [32], with low ratings primarily due to limitations in design as well as imprecision of results.

**Table 1:** Summary of included studies that focused on improving adherence in adolescents with CHCs using text message or mobile phone app interventions.

Source (condition)	Intervention (study design)	Age (years)	Participants	Adherence measure	Grade
Boker et al 2012, USA (acne) [34]	Text messages (RCT)	Mean (SD) (range): Entire cohort 22.7 (5.7) (12-35) Text 22.8 (5.6) (14-35) Control 22.5 (5.9) (12-32)	Total N: Enrolled (40) Lost follow-up (7) Final N=33: Intervention (15); Control (18)	Medication event monitoring system	Low
Fabbrocini et al 2014, Italy (acne) [39]	Text messages (RCT)	Mean age: Text 19.5 Control 18.5 Entire cohort range (14-28)	Total N: Enrolled (160) Lost follow-up (15) Final N=145: Intervention (74); Control (71)	7-day recall self-report	Low
Ostojic et al 2005, Croatia and USA (asthma) [47]	Text messages and in-person sessions (RCT)	Mean (SD): 24.6 (6.5)	Final N=16: Intervention (8); Control (8)	Self-report of daily medication use in a paper diary	Low
Hammonds et al 2015, USA (depression) [42]	Mobile phone app (RCT)	Mean (SD): 20.6 (4.3)	Total N: Enrolled (57) Lost follow up (10) Final N=40: Intervention (20); Control (20)	Pill count	Low
Cafazzo et al 2012, Canada (diabetes mellitus) [35]	Mobile phone app “ *bant”* (pilot trial)	Mean (SD): 15.1 (1.3)	Total N=20: Withdrawal (2) Incomplete data (6) Final N=12, Intervention	Self-report	Moderate
				Laboratory marker (glycosylated hemoglobin “HbA1c”)	
Franklin et al 2006, UK (diabetes mellitus) [40]	Text messages “Sweet Talk” (ST) (RCT)	Age (median, range): CIT 12.7 (10.5-14.8) Conventional insulin therapy /ST 14.1 (11.7-15.6) Intensive insulin therapy/ST 12.6 (11.2-15.4)	Total N: Enrolled (92) No allocation (3) Lost follow-up (1) Discontinued text (13) Final N=90: Conventional insulin therapy (27) Conventional insulin therapy and ST (32) Intensive insulin therapy and ST (31)	Self-report	Moderate
				Laboratory marker (HbA1c)	
Louch et al 2013, UK (diabetes mellitus) [43]	Text messages (RCT)	Range for entire cohort is 18-30	Total N: Enrolled (19) Lost follow-up (1) Final N=18: Intervention (8); Control (10)	Self-report	Low
Mulvaney et al 2012, USA (diabetes mellitus) [46]	Text messages “SuperEgo” (pilot trial with a historical control)	Mean (SD): Intervention 15.9 (2.9); Controls 15.8 (2.7)	Enrolled (28) Incomplete data (5) Final N=46: Intervention (23); Historically matched controls (23)	Laboratory marker (HbA1c)	Low
Dowshen et al 2012, USA (human immunodeficiency virus “HIV”) [37]	Text messages (pilot trial, pre-post design)	Mean (range): 23 (14-29)	Total N: Enrolled (25) Lost follow-up (3) Technical issue (1) Final N=21	Self-report (visual analogue scale and AIDS Clinical Trials Group)	Low
				Laboratory markers (viral load and CD4 cell count)	
Garofalo et al 2015, USA (HIV) [41]	Text messages (RCT)	Mean (SD): 24.1 (2.9) Median (range): 23 (18-29)	Final N=105: Control (51) Intervention (54)	Self-report (visual analogue scale)	Moderate
				Laboratory markers (viral load and CD4 cell count)	
McKenzie et al 2015, USA (liver transplant) [44]	Text messages (pilot trial with a historical control)	Median (range): 16 (12-20)	N=42	Laboratory testing participation rate	Low
Miloh et al 2009, USA (liver transplant) [45]	Text messages (pilot trial, pre-post design)	Median (range): 15 (1-27)	Total N: Enrolled (41) Dropout (17) Final N=24	Laboratory markers (tacrommilus levels and SD values)	Low
Creary et al 2014, USA (sickle cell disease) [36]	Text messages and Mobile Direct Observed Therapy “m-DOT” (pilot trial, pre-post design)	Mean (SD): 13.7 (6.3)	Total N=15 Final N=14	Observed adherence	Very low
				Self-report (Morisky Medication Adherence Scale)	
				Medication possession ratio	
				Laboratory markers (fetal hemoglobin and mean corpuscular volume)	
Estepp et al 2014, USA (sickle cell disease) [38]	Text messages “SIMON” (retrospective study)	Median (range): 13.9 (12.1-16.1)	Total N=83 Final N=55	Medication possession ratio	Low
				Laboratory markers (fetal hemoglobin and mean corpuscular volume)	
Ting et al 2011, USA (systemic lupus erythematosus) [48]	Text messages (RCT)	Mean (SD): 18.6 (2.5)	Final N=41: Intervention (19); Control (22)	Self-report (Medication Adherence Self-Report Inventory)	Low
				Medication possession ratio	
				Laboratory markers (hydroxychloroquine levels)	

### Intervention Type

The majority of interventions used text messaging to promote medication adherence via reminders or motivational messages (12/15, 80%) [34,37-41,43-46,48]. Of these 12 studies, only one combined text messages with other in-person intervention components (educational sessions) [47]. Additional interventions used other mobile phone‒based approaches or apps (3/15, 20%), including multifunction mobile phone apps [35,42] and mobile phone‒based directly observed therapy [36].

### Intervention Characteristics

#### Text Messaging Interventions

Text messaging interventions varied by frequency of messaging, message content, and added functionality. Most of text messaging interventions included one [36-38,40-43,45], two [34,39,48], or three [47,48] daily text reminders, or even more frequently in relation to meals [46]. Other studies provided reminders at different frequencies including weekly [40,41,46,47]; monthly, bi-monthly, or quarterly for laboratory monitoring [44]; and 1, 3, and 7 days before scheduled clinic appointments [48]. Most studies sent text message reminders that were customized to the patient’s medication regimen or personal preferences in terms of both scheduling (ie, time/day) and message content [34,37-41,43-48]. In addition, four studies had patients and/or parents create the content of the text messages themselves [38,40,45,46], and two of these studies included the use of a text message pool or bank [40,46]. The most sophisticated intervention designs also included messages related to dosing, side effects, adherence barriers, goal setting, positive reinforcement [39,40,43,46], or targeted to theoretical constructs [43]. Additional functionalities included patient reporting of physiological information via text (eg, peak expiratory flow in asthma patients) [47]; prompting of text-back responses from patients (two-way text messaging approach) [34,37,41,44,45]; sending text messages to parents if patients did not respond to scheduled reminders [45]; and the ability to request messages from individuals within their social network via the intervention platform [35,46] or from a motivational support network [40]. None of the text message intervention studies included a reward system or scheduled incentives for participants. Table 2 describes the approach and the components of different text messaging interventions.

**Table 2:** Description of text message interventions.

Author/year (condition)	Intervention purpose	Intervention description
Boker et al, 2012 (acne) [34]	Improve adherence to recommended use of topical acne medication (text messages)	Text messages twice daily (Duac in AM, Gifferin in PM) for 12 weeks
		Customized electronic reminder schedule at a specific time based on patient preferences and anticipated time of each medication use
		2-way communication: patients asked to text back a reply if and when each application was completed
		Identical text with general content to all patients, varied only by starting with patient’s first name
		Texts were sent through: www.LetterMeLater.com
Fabbrocini et al, 2014 (acne) [39]	Improve adherence to acne medications (text messages)	Texts twice daily for 12 weeks (11 consecutive days)
		Texts focused on frequently asked questions about acne medications, such as administration, daily dose, and side effects
		Texts were identical to all patients (no customization) covering 11 frequently asked questions
		Texts re-sent in same sequence every 11 days until end of 12 weeks
Ostojic et al, 2005 (asthma) [47]	Improve adherence to inhaled medications and peak expiratory flow (PEF) monitoring (text messages and in-person sessions)	Patients sent their PEF results daily via text messages for 16 weeks
		Data connected to a computer with software that automatically computed maximal, minimal, and mean PEF, PEF variability, and compliance
		PEF measurements 3 times daily with medication use and symptoms in paper diary
		Therapy was adjusted weekly by an asthma specialist according to peak expiratory flow meter (PEFM) values received daily from the patients
		1-hour asthma education session with specialist at clinic: discussed symptoms, asthma symptom score, indicators of control, medication use, and correct use of metered dose inhaler and PEFM
Louch et al, 2013 (diabetes mellitus) [43]	Improve insulin administration in young adults with type 1 diabetes; test moderation of intervention effect by personality factors (text messages)	Text messages sent daily (1-way communication) at 10 am for 14 days
		No customization of message content
		Text content was related to the correct insulin administration
		Text targeted constructs of the Theory of Planned Behavior: attitudes, subjective norms, perceived behavioral control, and intention
Mulvaney et al, 2012 (diabetes mellitus) [46]	Motivate patients and remind them with their diabetes self-care tasks (text messages “SuperEgo”)	8-12 text messages/week for 12 weeks
		Scheduled just before and after mealtimes and before bedtime
		Customization with users able to alter timing and frequency of messages through a website
		Messages could be scheduled in a 1-way communication at specific times of day within 15 minute increments and automated to be sent once only, or repeated based on participant preferences, such as daily, weekly, or on weekends
		Individually tailored messages: 75% of messages tailored to the top 3 patient-specific adherence barriers reported; and 25% of messages were randomly selected from the remaining message pool
		Four functions were included in the system: assessment, message selection, message scheduling, and requests for messages from others
		Text messages were created in collaboration with 96 adolescents with diabetes mellitus and no messages were repeated
		Participants could add their own messages, search for messages, and delete, change, or reschedule them using their mobile phone
		Participants could also search for and select messages that were associated with a particular goal
		Participants could ask other SuperEgo users for messages relating to a specific goal and then schedule that message for themselves
		Messages could be specified as private or public
		Participants could nominate people as part of their support team by entering that person’s email address into the system to contribute messages for patients
Franklin et al, 2006 (diabetes mellitus) [40]	Improve patients’ self-efficacy and glycemic control, and enhance their uptake of intensive insulin therapy (text messages “Sweet Talk”)	Text message reminders for 12 months
		Weekly reminders of the goal set in clinic, and daily reminders providing tips, information, or reminders to reinforce this goal
		Text messages automated, scheduled, and designed to offer regular support and optimize their self-management and control
		Database of over 400 messages that encompass the four main diabetes self-management tasks (insulin injections, blood-glucose testing, healthy eating, and exercise)
		Messages tailored based on patients goals and patients’ age, sex, and diabetes regimen
		Occasional text “newsletters” regarding topical diabetes issues
		Motivational support network
Dowshen et al, 2012 (HIV) [37]	Improve adherence to antiretroviral therapy among youth (text messages)	Daily 2-way text messages for 24 weeks
		Delivered at prespecified time schedule
		Personalized content, patients were encouraged to develop messages that maintain their confidentiality
		Interactive with follow-up messages with patients responding with number (1) if they took their medication and (2) if they did not
		Participants could reach out to study coordinator at any time to change the message or to reprogram the message if their mobile service was interrupted
		Texts were sent through: http://www.intelecare.com/
Garofalo et al, 2015 (HIV) [41]	Improve adherence to antiretroviral therapy among poorly adherent youth (text messages)	Daily text messages reminder for 6 months
		Initial messages were followed by a second message 15 min later to check if patients took their medications in a 2-way communication
		Personalized by subject for both content and schedule to be timed with medication doses
		Customization with initial message and follow-up messages were designed by the youth themselves
		Texts content were culture and identity sensitive and meaningful to participants
		Texts content used more indirect language to maintain confidentiality
		Motivational or encouraging follow-up messages were randomly sent to participants based on their affirmative or negative response
		Participants were encouraged to delete messages after taking medication and to use passcode protection to maintain phone confidentiality
		Sent/received and failed/invalid messages were summarized in weekly reports and sent to research staff to follow up with participants
		Texts were sent through: http://www.remedyhealthmedia.com/
Miloh et al, 2009 (liver transplant) [45]	Improve adherence to immunosuppressant medications (text messages)	Mean duration of the study 13 (SD 1.5) months
		Text schedule was customized at day/time specified by user
		2-way communication where patients were expected to respond to text message to confirm medication intake; if no response within 15 min to 1-hour caregiver notified via text
		Text messages were sent to the person responsible for medication intake (patient or caregiver)
		Patients or their caregivers registered and entered their information into texting platform (MediM system) with a personal password
		Entered information included patient’s name and mobile phone number, caregiver’s name/ nickname and mobile phone number, the medication name and frequency, and the exact times of text messages they want to receive
		Participants did not enter medication dose as that might change based laboratory test results
		No customization as text messages content was the same for all patients
		Text messages were read: “Take [name of medication] at [set time]. To confirm intake, press REPLY, type CARE 1, and press SEND.”
		Participants reimbursed for all text messages costs during the study
		Texts were sent through: http://www.carespeak.com/corp/
McKenzie et al, 2015 (liver transplant) [44]	Improve participation in laboratory testing among youth (text messages)	Automated laboratory tests text message reminders for 12 months
		Text message timed with lab tests (monthly, bimonthly, quarterly) as reminder to complete lab tests
		Text message reminders sent first Monday of each month when testing was due
		On last Monday of the month, patients received a message about laboratory testing completion
		Same message content for all patients
		2-way communication as patients replied back as yes/no responses
		No reimbursement for the cost of text messages, but all participants had unlimited text plans
		Mobile phone numbers entered into a secure website with secure-password
		$31/month to maintain the intervention or website domain
Estepp et al, 2014 (sickle cell disease) [38]	Improve adherence to hydroxyurea therapy (text messages: SIMON)	Scheduled daily text message reminders for 12 months
		Customizable for content, frequency, and duration
		Participants created their own messages
		Changes in text messages regimen checked every 3-4 clinic visits
		Messages delivery was monitored (received and undelivered) and participants could optionally reply
		Messages sent through a Web-based app
Ting et al, 2011 (systemic lupus erythematosus) [48]	A. Visit adherence intervention Improve adherence to scheduled clinic visits (text messages)	Text reminders sent 7, 3, and 1 day prior to each scheduled f/u clinic appointment
		Mean duration of the study 12 (SD 5) months
		Content was individualized for each patient and included the scheduled appointment time
		If a patient didn’t schedule follow-up appointment within 2–3 weeks after completed clinic visit, they would get a text reminder to do so
	B. HCQ adherence intervention Improve adherence to use of hydroxychloroquine (text messages)	Standardized daily text reminders for hydroxychloroquine intake daily or twice daily
		Mean duration of the study 12 (SD 5) months
		Text reminders at set time of day, according to hydroxychloroquine schedule
		Printed information sheet that was given to the standard of care group

#### Mobile Phone App Interventions

Mobile phone‒based interventions included native apps for delivery of medication reminders [42]; a multifunction app that includes integration of a wireless device for physiological measurement and visualization (ie, glucometer); self-monitoring alerts and prompts for gamification features to incentivize engagement with the app, with a secure network for peer communication [35]; and a multifunction app that includes daily alert messages, creation and sharing of patient videos to directly observe adherence to therapy with feedback/follow-up, positive feedback/reinforcement messages, and incentives for adherence [36]. Table 3 describes the approach and the components of different mobile phone app interventions.

### Study Outcomes

Of the 15 studies reviewed, 7 (47%) demonstrated statistically significant differences in medication adherence or related health outcomes [36,37,41,43-45,47]. The majority of the studies included in this review provided enough information to calculate standardized outcomes, such as SMDs. Most studies reported overall moderate to large SMDs of subjective and objective markers of adherence; however, most SMDs had wide 95% confidence intervals (Table 4). Several studies combined data related to the assessment of the efficacy or the usability/feasibility of different interventions reporting high levels of satisfaction and few technical or feasibility problems [35-37,40,41,44-46]. Additional results of each individual study are summarized in Multimedia Appendix 3 for text message interventions, and Multimedia Appendix 4 for mobile phone app interventions.

**Table 3:** Description of mobile phone app interventions.

Author/year (condition)	Intervention purpose	Intervention description
Hammonds et al, 2015 (depression) [42]	Improve adherence to antidepressant medications among college students using mobile phone app reminders	Medication reminders through mobile phone app for 4 weeks
		Entered prescribed information for medication doses
		Patients indicate when they had taken their medication by responding to reminders received
Cafazzo et al, 2012 (diabetes mellitus) [35]	Improve self-management among youth (mobile phone app “ *bant* ”)	App exposure for 12-week pilot study
		Adapter that allows a OneTouch UltraMini glucometer to communicate via Bluetooth, allowing the transfer of blood glucose reading wirelessly, to the iPhone device running the mobile phone app, “ *bant* ”
		Analysis tools assess the data soon after transfer to give adolescents real-time feedback
		Data analysis and trending screens display the percentage of blood glucose levels that are in range at specific times
		Communication with peers in an app-secure community area as a support network
		Rewards algorithm with point allocations
Creary et al, 2014 (sickle cell disease) [36]	Improve adherence to hydroxyurea (m-DOT, multidimensional strategy for 6 months)
		Alert reminders: automated daily alerts to remind patients to take hydroxyurea; alert sent at time preferred by patients; alerts stopped when a video is submitted; up to 4 text messages and email were sent daily
		Videos: participants created daily videos of them taking hydroxyurea; videos submitted electronically to the secure study website; captured by mobile phones or computers; included participants’ study ID; self-recorded videos for children 12 years or older, younger patients had assistance from parents
		Feedback: submitted videos were reviewed by research team within 72 hours of receipt; text and email feedback was sent to participants if they missed ≥2 video submissions in a 30-day period; participants were called if they missed ≥3 video submissions in a 30-day period; positive reinforcement (≥2 text messages or emails) was provided if they participants had adherence of ≥90%
		Incentives: if participants achieved ≥90% of adherence to hydroxyurea for each 30-day period, they would receive $1/day

## Discussion

### Principal Findings

Medication nonadherence is a widespread problem in pediatric CHCs, and among adolescents in particular. In this systematic review, we identified 15 studies that met all our inclusion criteria. Our results suggest that there is modest evidence to support the efficacy of text messaging and mobile phone apps as interventions to improve medication adherence in adolescents with CHCs. Most of the included studies were of low to moderate quality because of methodological limitations, imprecision of results, or both. The included studies showed evidence of the acceptability and feasibility of text messaging and mobile phone app interventions, suggesting a potentially promising area of intervention development and further investigations in the near future to better understand their efficacy and cost-effectiveness.

Our findings suggest moderate SMDs for most included interventions, which is consistent with earlier reports of adherence-enhancing interventions (ie, non-technology specific). However, given the heterogeneity of the included studies, the observed moderate effect size should be interpreted with caution [18,23]. In contrast, Pai and McGrady reported small effect sizes in a systematic review of adherence-promoting interventions [20]. In our review, the quality of the included studies was low to moderate, which was similar to a recent review of findings for technology-mediated interventions for treatment adherence across all age groups [19], and more specifically for adolescents with chronic physical conditions [28].

**Table 4:** Effect size *d* for the main outcomes of included studies.

Source (intervention)	Study adherence outcomes	*d* (95% CI)^a^
Boker et al, 2012 (text messaging) [34]	Medication event monitoring system	No data available
Fabbrocini et al, 2014 (text messaging) [39]	7-day recall self-report	No data available
Ostojic et al, 2005 (text messaging and in-person sessions) [47]	Self-report of daily inhaled corticosteroids	0.35 (-0.64 to 1.4)
	Self-report of daily beta2-agonist	0.7 (-0.31 to 1.71)
Hammonds et al, 2015 (mobile phone app) [42]	Pill count of antidepressants	0.31 (-0.21 to 0.83)
Cafazzo et al, 2012 (mobile phone app) [35]	Self-report using personal blood glucose meters	0.11 (-0.69 to 0.91)
	Laboratory markers using glycosylated hemoglobin	0.45 (-0.36 to 1.26)
Franklin et al, 2006 (text messaging) [40]	Self-report using a visual analogue scale	0.38 (-0.14 to 0.89)
	Laboratory marker using glycosylated hemoglobin	0.12 (-0.4 to 0.63)
Louch et al, 2013 (text messaging) [43]	Self-report of insulin administration	1.1 (0.11 to 2.1)^b^
Mulvaney et al, 2012 (text messaging)	Laboratory markers using glycosylated hemoglobin	0.5 (-0.1 to 1.1)
Dowshen et al, 2012 (text messaging) [37]	Self-report using visual analogue scale	1.43 (0.75 to 2.11)^b^
	Self-report using AIDS Clinical Trials Group questionnaire	0.86 (0.22 to 1.49)^b^
	Laboratory marker using viral load	0.43 (-0.18 to 1.04)
	Laboratory marker using CD4 cell count	0.19 (-0.42 to 0.79)
Garofalo et al, 2012 (text messaging) [41]	Self-report using visual analogue scale	0.49 (0.11 to 0.89)^b^
	Laboratory markers using viral load	0.19 (-0.18 to 0.58)
McKenzie et al, 2015 (text messaging) [44]	Laboratory testing participation rate	0.66 (0.22 to 1.1)^b^
Miloh et al, 2009 (text messaging) [45]	Laboratory markers using tacromilus – overall	1.23 (0.62 to 1.85)^b^
	Laboratory markers using tacromilus – one medication	1.02 (0.42 to 1.6)^b^
	Laboratory markers using tacromilus – two medications	1.39 (0.77 to 2.03)^b^
	Laboratory markers using tacromilus – three medications	2.11 (1.41 to 2.82)^b^
Creary et al, 2014 (text messaging and m-DOT) [36]	Medication possession ratio	1.04 (0.25 to 1.83)^b^
	Laboratory markers using fetal hemoglobin	0.1 (-0.31 to 0.51)
	Laboratory markers using mean corpuscular volume	0.54 (-0.22 to 1.29)
Estepp et al, 2014 (text messaging) [38]	Medication possession ratio	0.07 (-0.31 to 0.44)
	Laboratory markers using fetal hemoglobin	0.1 (-0.31 to 0.51)
	Laboratory markers using mean corpuscular volume	0.18 (-0.24 to 0.59)
Ting et al, 2011 (text messaging) [48]	Self-report using medication adherence inventory	No data available
	Medication possession ratio	No data available
	Laboratory marker for hydroxychloroquine	No data available

^a^Positive effect size value means improvement in a study outcome, while a negative one means worsening outcome.

^b^Statistically significant *P*<.05.

While text messaging and mobile phone app interventions offer a straightforward approach to address adherence to medications among adolescents, with broad application across CHCs, some challenges exist. None of the included studies measured the long-term durability of intervention effects across randomized conditions; thus, there is a need to establish the exposure or dosage needed to sustain long-term effects. In addition, the characteristics of the included studies also suggest that there is a need for improvement in intervention design; only four included theoretical models or approaches to target specific mechanisms of action to optimize efficacy [35,40,41,43]. McGrady and colleagues have recommended that articulation of the underlying mechanism of action for adolescent-specific adherence interventions is an advancement much needed to bring developmental and behavioral specificity to this growing area of research [50]. Furthermore, only two studies measured potential moderators of the intervention effect [41,43].

Several of the studies measured feasibility and acceptability outcomes and found high levels of satisfaction and few feasibility issues, which are promising for advancements in these technologies and consistent with earlier reports [19,21,28]. The high level of satisfaction is noteworthy given the frequency of messaging, which for most studies included at least daily messages, reflecting a relatively high tolerance in this group for intervention. The evidence of feasibility in these studies suggests that adolescent-specific text messaging and mobile phone‒based approaches may be an important and promising area of future intervention development. Given methodological limitations in the studies reviewed, larger studies with long-term outcomes are warranted, particularly with sufficient power for clinically important outcomes. Recent evidence supports the efficacy of text message and mobile phone app interventions to promote preventive behavior among adolescents [51]. Furthermore, in addition to efficacy data, cost-effectiveness is another important aspect of intervention evaluation [9,22,52]. The cost to develop and maintain each intervention can be a barrier to widespread use of these interventions. Cost also impacts the variability in patients’ access to technologies. Formal economic evaluation of different interventions will inform health care authorities to decide whether adoption of such interventions would be economically efficient [22,52]. In a related systematic review, we found insufficient evidence regarding the cost-effectiveness of text messaging and mobile phone apps to promote adherence in adolescents with CHCs [52]. Our findings highlight the need for further investigation of cost-effectiveness to inform the scalability, sustainability, and future cost savings of such approaches [52].

### Strengths and Limitations

Our systematic review has a number of strengths. First, we conducted our review following the recommendations for rigorous systematic reviews methodology [32,49,53]. Second, we used a highly sensitive search strategy guided by a librarian information specialist and had no language restrictions in order to minimize publication bias by identifying as many relevant studies as possible. Additional resources were also searched including published systematic reviews, clinical trial registries, and different electronic databases. Third, although we limited our search to studies published since 1995, there were no eligible studies identified before 2005. Therefore, the possibility that we have missed earlier studies is very small. Finally, 2 authors completed the review process independently at all stages.

Our systematic review of the literature should be considered within the context of some potential methodological limitations. First, similar to any other systematic literature review, although our search criteria were designed to be comprehensive, it is possible that we missed relevant articles. Second, we included only articles published in peer-reviewed journals, and publication bias with the tendency to report positive study results cannot be excluded [54]. Third, a number of studies reported insufficient information, the definition of adherence varied, and the study sample size and ages as well as methods of adherence assessment used in the included studies were heterogeneous. These limitations prohibited a meta-analysis from being performed [55]. Finally, many of the included studies had relatively small sample sizes.

### Conclusions

The number of adolescents with CHCs continues to increase and medication nonadherence is a clear challenge. The use of text messaging and mobile phone app interventions to improve medication adherence among adolescents with CHCs has shown promising feasibility and acceptability; however, there is modest evidence to support their efficacy. Further evaluation of short- and long-term efficacy and cost-effectiveness of these interventions is warranted. In addition, better understanding of barriers to medication adherence would inform further development of text message and/or mobile phone app interventions to improve adherence and health outcomes in adolescents with CHCs, such as sickle cell disease [56,57]. Adolescents are frequent users of text messaging and mobile phone apps, and engaging adolescents with CHCs in their self-management is critical for improving long-term outcomes. Seeking adolescents’ perspectives could enhance uptake and long-term engagement, while minimizing patient fatigue. The currently available data from medication adherence studies using text messaging and mobile phone app interventions are heterogeneous, particularly in relation to process and outcomes measures, which limit the evidence generated and conclusions that can be drawn from those studies. Nevertheless, consistent use of Web-based and mobile health interventions reporting guidelines [58] would enhance comparative research across studies [59]. The functionalities of different mobile technologies continue to improve with gradually decreasing cost suggesting potential economies of scale where interventions could be delivered to large populations.

## Appendix

Multimedia Appendix 1 PRISMA checklist.

Multimedia Appendix 2 Search strategies.

Multimedia Appendix 3 Text message intervention outcomes.

Multimedia Appendix 4 Mobile phone app intervention outcomes.

## Abbreviations

CHCs: chronic health conditions
GRADE: Grades of Recommendation, Assessment, Development, and Evaluation
HRQOL: health-related quality of life
m-DOT: Mobile Direct Observed Therapy
PEF: peak expiratory flow
PEFM: peak expiratory flow meter
PRISMA: Preferred Reporting Items for Systematic Reviews and Meta-Analyses
PROSPERO: International Prospective Register of Systematic Reviews
SMD: standardized mean differences
