# Supplementary material for: Text Messaging and Mobile Phone Apps as Interventions to Improve Adherence in Adolescents With Chronic Health Conditions: A Systematic Review
Source: JMIR Mhealth Uhealth. 2017 May 15;5(5):e66. doi: 10.2196/mhealth.7798 (PMC5447825; doi:10.2196/mhealth.7798)
Supplement: Multimedia Appendix 3 [file mhealth_v5i5e66_app3.pdf]

Multimedia Appendix 3: Outcomes of text message interventions

Author/year (Condition)	Study Outcomes
Boker et al, 2012 (Acne) [34]	**Adherence:**<br>• Mean adherence for both daily medications over 12 weeks: 33.9% in text-group vs. 36.5% in controls (p=0.75)<br>• Average self-reported adherence rate in text-group was 128.5 doses (74.4%)<br>• In the entire cohort, patients with higher adherence rates had greater decrease in lesion count at week 12 (non-significant, NS)<br><br>**Clinical:**<br>• Investigator Global Assessment scores (baseline vs. 12 wk): 2.3 vs. 1.2 (-1.07) in text-group; 2.4 vs. 1.6 (0.68) in controls, (p=0.37)<br>• Proportion of patients with ''clear'' or ''almost clear'' Investigator Global Assessment score at week 12: 73.3% in text-group vs. 50% in controls (p=0.19)<br>• Mean self-reported improvement of acne severity by PGS: 55.3% in text-group vs. 57.5% in controls (NS)<br>• Total acne lesion count reduction (baseline vs. week 12), by Investigator Global Assessment: 66.6% in text-group vs. 53.4% in controls (p=0.21)<br>• Dermatology Quality of Life Index scores (baseline vs. week 12): 55.7 ± 22.6 vs. 96 ± 15.9 in text-group; 53.8 ± 26.8 vs. 94.3 ± 18 in controls (NS)
Fabbrocini et al, 2014 (Acne) [39]	**Adherence:**<br>• Adherence (baseline vs. 12 wk): 4.1 vs. 6.6 days/wk in text-group; 4.3 vs. 4.9 days/wk in controls, (p <0.0001)<br><br>**Clinical:**<br>• Global Acne Grading system scores (baseline vs. 12 wk): 25.3 ± 8.9 vs. 8.7 ± 3.6 in text-group; 24.7 ± 7.6 vs. 16.2 ± 5.6 in controls, (p <0.0001)<br>• Dermatology Quality of Life Index scores (baseline vs. 12 wk): 9.2 ± 2.2 vs. 5.4 ± 1.8 in text-group; 9.5 ± 1.8 vs. 8 ± 1.4 in controls, (p <0.0001)<br>• Cardiff Acne Disability Index scores (baseline vs. 12 wk): 8.6 ± 1.3 vs. 2 ± 0.8 in text-group vs. 7.8 ± 1.2 vs. 5.1 ± 0.8 in controls, (p <0.0001)<br>• Patient-Doctor Depth-of-Relationship Scale scores (baseline vs. 12 wk): 15 vs. 25 in text-group, 11 vs. 18 in controls.<br>• Most patients were satisfied with the text-group (65% very and 30% quite satisfied)
Ostojic et al, 2005 (Asthma) [47]	**Adherence:**<br>• No significant difference among study groups in daily consumption or adherence of inhaled medicine (steroids: 625 ± 332 vs. 530 ± 200 micg, p=0.574; B2-agonists: 118 ± 63 vs. 84 ± 28 micg, p=0.383<br><br>**Clinical:**<br>• Peak expiratory flow (PEF) variability, mean (standard deviation, SD): 16.12 ± 6.93% in text-group; 27.74 ± 10.01% in controls (p=0.049)<br>• No significant difference among study groups in forced vital capacity<br>• Mean forced expiratory volume in first second (FEV1) was similar in the two groups before and after the study<br>• FEV1, mean (SD): 81.25 ± 17.31 in text-group vs. 77.63 ± 14.80 in controls (p=0.014)<br>• Cough symptoms (0-3, 3 being worst): 1.42 ± 0.28 in text-group vs. 1.85 ± 0.43 in controls (p<0.05)<br>• Night symptoms (0-3, 3 being worst): 0.85 ± 0.32 in text-group vs. 1.22 ± 0.23 in controls (p<0.05)
Louch et al, 2013 (Diabetes Mellitus) [43]	**Adherence:**<br>• Intervention significantly improved evening injection rates only in the low conscientiousness and low Conscientiousness and consideration of future consequences (CFC) groups (no additional details available)

	• Patients with high conscientiousness showed few differences in evening injection rates across groups<br>• Patients with low conscientiousness showed clear differences across groups<br>• Significant interactions of condition with conscientiousness (p=0.001), CFC (p=0.007) and a 3-way interaction among condition, conscientiousness, and CFC (p=0.009)
Mulvaney et al, 2012 (Diabetes Mellitus) [46]	**Adherence:**<br>• At 3 months, the mean glycosylated hemoglobin (HbA1c) level in the intervention group was unchanged (8.8%), but the mean level in the control group was higher (9.9%), p=0.006<br><br>**Usability/Acceptability:**<br>• Website log-ins average 3/wk (range 1-8)<br>• Average messages:<br>o Received 10/wk (range 8-12)<br>o Newly created 2.9 (SD 2.7)<br>o Additionally scheduled 5.0 (SD 4.2)<br>o Deleted 1.8 (SD 0.9)<br>• 9 people (friend or family) were nominated by participants to contribute messages to help with diabetes<br>• 33% of new messages focused on new reminders and motivation for blood glucose monitoring<br>• System usability and satisfaction were rated highly<br>• There was a significant main effect of time (p=0.02), no significant main effect of group (p=0.42), and a significant interaction between group and time (p=0.006)
Franklin et al, 2006 (Diabetes Mellitus) [40]	**Adherence:**<br>• Self-reported adherence scores: 70.4 ± 20.0 in Conventional insulin therapy (CIT) alone vs. 77.2 ± 16.1 in CIT and Sweet Talk (ST) (95% CI +0.4, +17.4, p=0.042)<br>• Mean glycemic control improved in intensive insulin therapy (IIT) and ST (9.2 ± 2.2%, 95% CI −1.9, −0.5, P < 0.001), compared to CIT and ST<br>• Self-efficacy for diabetes scores: 56.0 ± 13.7 in CIT alone vs. 62.1 ± 6.6 in CIT and ST (95% CI +2.6, +7.5, p=0.003)<br><br>**Usability/Acceptability:**<br>• Patients' perception of the quantity of support they received from the diabetes team was higher in ST groups, but not support from family and friends<br>• Sweet Talk system feedback:<br>o 81% helpful for DM self-management<br>o 90% wanted to continue text messages<br>o 97% liked messages frequency (1-2 daily) 20% disliked repeated similar messages
Dowshen et al, 2012 (Human Immunodeficiency Virus) [37]	**Adherence:**<br>• Mean visual analogue scale (VAS) scores (0-100, with 100 being most adherent): 74.7 (wk 0) vs. 93.3 (wk 12), (p<0.001); 74.7 (wk 0) vs. 93.1 (wk 24), (p<0.001)<br>• AIDS Clinical Trials Group questionnaire 4-day recall (0-4, with 4 being most adherent): 2.33 (wk 0) vs. 3.24 (wk 12), (p=0.002); 2.33 (wk 0) vs. 3.19 (wk 24), (p=0.005)<br>• CD4 cell count or viral load (wk 0 vs. wk 12 or 24-wk: a trend toward improvement with a small to moderate effect size (Cohen d: –0.51 to 0.22)<br><br>**Usability/Acceptability:**<br>• A total of 15,387 messages were sent through the Intelecare platform, 14,220 messages were successfully sent<br>• Of the 7110 messages requesting participants to respond, 3414 (48%) replied whether they took their medications or not<br>• 20/21 (95%) participants found texts helpful to avoid missing doses<br>• 17/21 (81%) participants wanted to continue to receive text after the study is completed
Garofalo et al, 2015 (Human Immunodeficiency Virus) [41]	**Adherence:**<br>• VAS adherence difference between intervention and controls (0 vs. 3 mon): 7% points (95% CI, 0.91, 13.9) (p<0.05); 2.57 OR for ≥90% adherence (95 % CI 1.01–6.54) (p<0.05)

	• VAS adherence difference between intervention and controls (0 vs. 6 mon): 3.5% points (95% CI, –2.03, 9.11) (NS); 2.12 OR for ≥90% adherence (95 % CI 1.01–4.45) (p<0.05)<br>• Intervention sustainability effect (VAS ≥90% during follow up: 58% at 9-month vs. 61% at 12-month (p=0.6)<br>• Intervention effect on VAS (≥90%) after cross-over (initial control group): 51% at baseline (6-month) vs. 65% at 12-month (p=0.07)<br>• No significant differences in either log viral load between the two study arms at either 3- or 6-month follow-up<br>• Participants with high levels of depression and marijuana use (32%) had significantly lower adherence compared to those with neither (p=0.005)<br><br>**Usability/Acceptability:**<br>• A total of 9,586 reminder messages sent, 8,512 were successfully received (89%)<br>• 58% responded to the reminders at least once<br>• 100% would recommend intervention for a friend on daily meds<br>• 81 % wanted to continue to receive text after the study is completed<br>• 95 % satisfied with intervention overall, supported by open-text comments
Miloh et al, 2009 (Liver Transplant) [45]	**Adherence:**<br>• Mean tacrolimus levels SD values (before vs. after): 3.46 ± 2.17 vs. 1.37 ± 1.01 mg/L (p<0.005)<br>• Number of patients with tacrolimus level SD values above threshold (SD >25) decreased from 24 to 6 (P=0.19)<br>• Mean Sirolimus level SD values (before vs. after): 5 vs. 1.8 g/L (p=0.01)<br>• Mean tacrolimus levels SD values during the study (before vs. after):<br>o One-medication regimen: 3.18 ± 2.35 to 1.27 ± 1.24 g/L (p<0.005)<br>o Two-medication regimen: 3.65 ± 2.10 to 1.45 ± 0.74 g/L (p<0.005)<br>o Three-medication regimen: 4.15 ± 1.62 to 1.61 ± 0.52 g/L (p=0.02)<br>• The mean age of patients: self-administration of medications (17.38 ± 4.06 years); Medications delivered through parents (7.75 ± 5.21 years) (p<0.005)<br><br>**Clinical:**<br>• Number of histologically proven, acute, cellular rejections (before vs. after): 12 vs. 2 episodes (p=0.02)<br><br>**Usability/Acceptability:**<br>• Compliance rates during study: 69% of patients completed the study<br>• 48% of patients dropped out<br>• No risk factors for dropout were identified
McKenzie et al, 2015 (Liver Transplant) [44]	**Adherence:**<br>• Laboratory testing participation rate (before vs. after): 58% (Mean=0.58, SD=0.31) vs. 78% (Mean=0.78, SD=0.30), (p<0.001)<br>• Laboratory testing participation rate (intervention vs. controls): at baseline, no difference (p=0.8); after study, high rates in intervention-group (p=0.003)<br><br>**Clinical:**<br>• 11 patients changed the rate of their laboratory frequency based on their clinical situation<br><br>**Usability/Acceptability:**<br>• Participants preferred secure email (32%) or text messaging (68%) as the primary was of medical communication

	• During the study period, all participants continued the intervention and no phone numbers were disconnected<br>• 30 patients (77%) responded with at least one text message during the study<br>• 12 patients (29%) communicated with additional messages, mainly to clarify a revised laboratory schedule<br>• Text messages sent:<br>o Average 5.7 ± 3.6 (range 1–13)/patient<br>o 95 "YES" (Mean 3.2 ± 2.8, range 0–11), 94% correctly completed lab test<br>o 70 "NO" (Mean 2.4 ± 2.4, range 0–11), 96% lab test not completed by chart review<br>o Agreement k=0.89, 95% CI=0.82, 0.96 (p<0.001)<br>• Intervention feedback:<br>o 80% positively responded and found it helpful<br>o 70% felt that reminders definitely or possibly "made them more likely to get laboratory tests"<br>o Only 3 patients had technical issues<br>o 71% wanted the reminders to continue after study period<br>o 97% reported that reminders could help at least one aspect of self-management
Estepp et al, 2014 (Sickle Cell Disease) [38]	**Adherence:**<br>• No significant improvement in medication possession ratio or hydroxyurea adherence before and after SIMON<br>• At the end of the study, participants had higher mean corpuscular volume, hemoglobin levels and fetal hemoglobin percentages, and lower absolute reticulocyte counts and bilirubin levels, suggesting improved adherence to hydroxyurea<br>• Participant's change in medication possession ratio over time was predictor of improved fetal hemoglobin percentages<br><br>**Clinical:**<br>• No significant differences in emergency room visits or hospitalization or prior to and after initiation of SIMON<br>• Outpatient visits:<br>o Median of 9 visits (inter-quartile range, 7–11) one-year post-SIMON initiation<br>o Median decrease of 1 visit (p=0.013), compared to year pre-SIMON
Ting et al, 2011 (Systemic Lupus Erythematosus) [48]	**Visit Adherence study:**<br>• Adherence rates: pre: 13/70 (19%) non-adherent, post: 7/70 (10%) non-adherent (p=0.01), and overall higher than baseline (p=0.005)<br>• Higher adherence to clinic visits with:<br>o White race (p=0.04)<br>o Non-Medicaid status (p=0.03)<br>o Increased distance from hospital (p=0.008)<br>• Patients with more frequent visits had:<br>o More frequent no-shows, ER visits, and hospital admissions<br>o Worse Systemic Lupus Erythematosus Disease Activity Index scores<br>o Were treated with more medications<br>• Number of no-shows to clinic correlated with the number of ER visits and hospital admissions across all time periods<br>• Patients with lower median family income had:<br>o More frequent cancellations (p=0.04)<br>o More hospitalizations (p = 0.01)<br>o Worse mean Systemic Lupus Erythematosus Disease Activity Index scores (p = 0.0008)<br><br>**Medication Adherence study:**<br>• Hydroxychloroquine blood levels correlated with adherence rates measured by pharmacy refills (r=0.5, p<0.0001) and Medication Adherence Self-Report Inventory (r=0.47, p<0.0001)<br>• Using Medication Adherence Self-Report Inventory, pharmacy refill, Hydroxychloroquine blood levels, text reminders had a small effect size (Cohen's d <0.25) on the adherence to Hydroxychloroquine with no difference in patients with daily or twice daily regimens

	• In text reminders group, there was no improvement in participants' disease activity
