# Supplementary material for: Text Messaging and Mobile Phone Apps as Interventions to Improve Adherence in Adolescents With Chronic Health Conditions: A Systematic Review
Source: JMIR Mhealth Uhealth. 2017 May 15;5(5):e66. doi: 10.2196/mhealth.7798 (PMC5447825; doi:10.2196/mhealth.7798)
Supplement: Multimedia Appendix 4 [file mhealth_v5i5e66_app4.pdf]

Multimedia Appendix 4: Outcomes of smartphone app interventions

Author/year (Condition)	Main Results
Hammonds et al, 2015 (Depression) [42]	**Adherence:**<br>• Participants in intervention group were 3.5 times more likely to adhere to medication regimen than control group (p=0.057)<br>• Adherence by pill count was not significantly different between groups<br>• Overall adherence: 45% adherent (>=80%), 40% under-users (<80%), and 6% over-users (>100%), all in controls<br><br>**Clinical:**<br>• Depression was not significantly different between groups<br>• Medication under-users of were 3.4 more likely to endorse illicit drug use
Cafazzo et al, 2012 (Diabetes Mellitus) [35]	**Self-management/Adherence:**<br>• Self-care inventory showed no significant changes in all dimensions, including adherence<br>• The frequency of daily average blood glucose measurements increased by 50% from 2.4 to 3.6 per day (p=0.006)<br>• Glycosylated hemoglobin (HbA1c) values did not significantly change over pilot period (p=0.11)<br><br>**Usability/Acceptability:**<br>• Study satisfaction was high, 88% stated that they would continue to use the system, and 50% of patients had more than 10 awards<br><br>**Clinical:**<br>• Diabetes HRQOL dimensions and parent–adolescent patient interactions showed no change over study-period
Creary et al, 2014 (Sickle Cell Disease) [36]	**Adherence:**<br>• Morisky Medication Adherence Scale (MMAS-4) scores (<2): 9/14 participants at 2–4 months and 10/14 participants at 6 months (p=0.004)<br>• Medication possession ratio adherence (median, IQR): pre-study 0.75 (0.59–0.82) vs. post-study 0.91 (0.85–1.00) (p=0.02)<br>• Observed HU adherence rate: median of 93.3% for each month, 10/14 had ≥90% adherence, and 12/14 had ≥80% adherence<br>• Mean corpuscular volume (median, IQR): pre-study 96 (91–107.9) vs. post-study 107.2 (96.3–113.3) (p=0.009)<br>• HbF (median, IQR): pre-study 10.5 (6–17) and post-study 11.4 (9.3–18.9) (p=0.03)<br>• Treatment Satisfaction Questionnaire for Medication (TSQM-9) in patients with MMAS-4 (<2) (mean, standard deviation): pre-study 82.8% ± 16.7% vs.ost-study 95.6% ± 5.1% (p=0.03)<br>• TSQM-9 in patients with medication possession ratio (≥90): pre-study 74.7% ± 16.6% vs. post-study 96.0% ± 5.3% (p=0.008)<br><br>**Usability/Acceptability:**<br>• Less than 20 minutes daily to complete study observations, record adherence, and provide feedback to the enrolled participants<br>• Mobile-DOT Trial period: 13/14 completed in <14 days and 1/14 completed in 30-days<br>• Text messages didn’t disrupt participants’ daily activities<br>• Mobile DOT was not intrusive<br>• 13/14 participants completed Mobile-DOT in <3 minutes daily<br>• All participants continued to submit videos and receive alerts, feedback, and incentives as part of extension study
